# Valuation of Estimation Toxic Chemical Release Inventory Method-Focusing on Paint Manufacturing Process

**DOI:** 10.3390/ijerph16183260

**Published:** 2019-09-05

**Authors:** Hyo Eun Lee, Eun-Hae Huh, Yi Yoon, Seok J. Yoon, Da-An Huh, Kyong Whan Moon

**Affiliations:** 1 Department of Health Science, Korea University, Anam-ro 145, Seongbuk-gu, Seoul 02841, Korea (H.E.L.) (E.-H.H.) (S.J.Y.) (D.-A.H.); 2 Accident Prevention and Assessment Division,90 Gajeongbuk-ro, Yuseong-gu, Daejeon 34111, Korea

**Keywords:** toxic release inventory, volatile chemical substance, paint manufacturing process, emission factor method, material balance method, source testing method, emission model method

## Abstract

Industrial chemicals differ in their treatment methods and types, depending on their physicochemical properties. Highly volatile chemicals are emitted despite installation of preventive facilities, such as scrubbers and adsorption towers. Some countries release a Toxic Release Inventory (TRI), which is a mandatory report on the amount of chemicals emitted annually. This report is released to the citizens to ensure their right to knowledge and life. Numerous methods have been devised to investigate the amount of chemical emissions. There are four methods to estimate TRI emissions (Emission Factor Method; Material Balance Method; Source Testing Method; Emission Model Method). Moreover, efforts have been made to increase awareness and formulate plans to reduce chemical emissions. Despite this, the TRI method tends to underestimate and overestimate, especially due to volatile compounds. If the results of the TRI emissions are underestimated, toxic chemicals can have a negative impact on citizens. Volatile compounds are commonly used in chemical manufacturing plants, such as paint plants. In this study, a suitable method for each industrial process was suggested based on conservative estimates of multiple toxic chemical inventory method, focusing on the paint manufacturing process. In the paint manufacturing plant, storage, weighing, and mixing processes should be used emission model method to estimate TRI. In the reaction process, TRI must be estimated by the source test method. In the transfer process, the emission factor method should be used to estimate TRI. In the atmosphere prevention process, the emission factor method or source testing method should be used depending on the physical and chemical properties such as vapor pressure of the chemical.

## 1. Introduction

Accidents that occur at chemical plants affect not just the surrounding environment (air, water, soil, etc.) but also humans. To date, many chemical accidents have occurred worldwide, leading to the creation of various safety management systems. In 1976, an accident occurred in Seveso, Italy, where chlorine gas and dioxin leaked into the atmosphere, causing damage to 11 nearby villages. This led to the enactment of the Seveso Directive. After the 1989 Philips plant explosion, the United States introduced the Process Safety Management regulations, which strengthened internal chemical safety management [1]. The MIC (Methyl Isocyanate) explosion in 1984 in Bhopal, India, and the 1984 explosion in the West Virginia chemical plant in the United States revealed the need for stringent and different regulations than just chemical plant safety controls. After these accidents, residents around the chemical plants formed an agreement to be made aware of the type and amount of chemicals handled at the nearby plants. The first Emergency Planning and Community Right-to-Know Act was enacted in the United States in 1986. Moreover, Toxicity Release Inventory (TRI) was established to disclose to local residents how much of the hazardous chemicals used in industrial sites are released [2].

Agenda 21, adopted by the United Nations Conference on Environment and Development (UNCED) in 1992 Brazil, included the management of hazardous chemicals and environmental safety (Chapter 19). In 1996, the Organization for Economic Cooperation and Development (OECD) recommended that Member States make recommendations on the Pollutant Released and Transfer Registration [3].

As a result, OECD countries around the world are investigating the release of chemicals and disclosing them to the public. Korea, that joined the OECD in 1996, has legislated guidelines for TRI, and included a provision to investigate emissions every year, according to Article 11 of the Chemical Control Act, as of 2019 [4].

TRI results are not simply disclosed to the public. Moreover, as the amount of chemicals released varies by region, there may be health inequalities among local residents. Studies on Texas have shown that women who live in areas with high levels of carcinogens are less likely to get pregnant than women who do not [5]. This shows that TRI is not just an annual report, but actually affects the health of local residents. This can be used as an indicator to protect the health of local residents through measures such as high emission factories. These results can be used to formulate policies to reduce the amount of carcinogenic chemical substances emitted by plants. Furthermore, the increase and decrease in the amount of chemical substances emitted by each region are important indicators for policy decisions with respect to health and local government [6].

TRI needs to be accurate and stringent. However, it is difficult to estimate the amount of chemical emissions from each chemical plant. Underestimation and overestimation in the current TRI emission survey system are factors that reduce the accuracy of the current emission survey system, such as the lack of understanding by the managers in the workplace, the content of difficult guides, the lack of error verification functions when entering data, and the lack of awareness of how to identify and manage the sources [7]. It is said that evaluation may be made. The pathway for the release of chemicals to the atmosphere is complex, making it almost impossible to estimate the amount accurately, particularly when there are leakages from pipes (non-point pollution source) or chimneys (point pollution source). Also, highly volatile chemicals are released into the atmosphere, making it difficult to estimate emissions [8].

Several methods have been developed to estimate TRI and are adapted based on the characteristics of the plant. In Korea, any one of the four methods, that is emission factor method, material balance method, source testing method and emission model method is selected for each process, and the amount of emissions (TRI) is calculated and submitted to local governments [9].

However, there is a large difference in the results obtained from each method. Moreover, the Korean local government focuses on the missing chemical substances in the chemical factory, rather than evaluating the feasibility of the method. This difference in TRI results can cause confusion for both the plant and the public. Some people risk exploiting this to report that they do not release much toxic chemicals. TRI needs clearer and more conservative guidance.

For this study, paint manufacturing plants were selected as they handle many volatile substances, such as solvents (toluene, xylene). Moreover, various TRI methods were evaluated for each paint manufacturing process to present the most conservative and stringent method.

## 2. Materials and Methods

### 2.1. Selection of Chemical Substances and the Discharge Route

When comparing the different chemical industries around the world with respect to the chemicals emitted, there are significant differences observed, depending on the type of industry that serves as the industry base of the country. In the case of the United States, the chemical manufacturing industry had the third highest total amount of TRI emissions, followed by electronics and metal manufacturing industries. In Japan, the chemical manufacturing industry ranked first, and in Australia, it ranked fourth after the electronic manufacturing, metal manufacturing, and mining industries. Korea’s chemical manufacturing industry ranked first, followed by metal manufacturing and electronics manufacturing industries (Table 1) [10]. Overall, the total amount of chemical substances emitted by chemical manufacturing industries in each country was substantially high.

The top five chemicals in the United States, Japan, Australia, and Korea were studied, excluding heavy metals and combustion-related compounds (carbon monoxide, nitrogen compounds, etc.) and their emission ratios were calculated (Table 1). In Korea and Japan, where the chemical industry is the largest sector, volatile organic compounds such as xylene and toluene are widely used. On the other hand, in the United States and Australia, where the electronic parts manufacturing industry is the largest sector, hydrochloric acid and methanol were the most used chemicals.

Chemical substances can be divided into water soluble chemicals, such as hydrochloric acid, sulfuric acid and ammonia water, and VOCs, such as toluene, xylene, methyl ethyl ketone, and dichloromethane. Chemicals that are in a liquid state can be treated with water or a solution and be easily collected. Moreover, if they are in a gaseous state, they can be treated with scrubbers for collection. However, even if VOCs are removed using activated carbon, the removal efficiency is low and it generates secondary pollutants, such as CO, NOx, and SOx, despite treatment with Regenerative Thermal Oxidizer (RTO) or flare stack. Non-point pollution sources originating from pipelines and tanks generate large amounts of emissions because of their high volatility [11]. In this study, three kinds of volatile substances, namely toluene, xylene and methyl ethyl ketone, which have the highest total emissions, were studied.

The route through which chemicals are released is divided into three categories: Air, water and soil. Most of these chemicals are released into the air. The emission path ratio of each country is shown in Table 2 [12]. In particular, emissions to the air are via point pollution sources and non-point pollution sources. Point pollution sources include facilities for preventing environmental pollution such as scrubbers, adsorption towers, RTO, and flare stack. In the case of point pollution sources, it is possible to monitor the amount discharged and to continuously manage emissions through measurement. However, non-point pollution sources include volatile substances in process flows, such as tanks or pipes; hence, management and estimation of the amount of discharge is difficult. In this study, we evaluate the feasibility of the emission estimation method of the point source pollution source by selecting the discharge route to the air.

### 2.2. Selection of Industries and Process

Toluene, xylene, and methyl ethyl ketone are volatile chemicals used in various chemical processes to dissolve other materials and in the manufacture of a variety of chemicals, from aviation fuel to automotive fuel plastics to paint.

Huge manufacturing plants have flow processes that are designed to reduce environmental pollution and to reduce direct exposure of workers in the manufacturing line to chemicals. Moreover, they use RTO or flare stack (air pollution control facility) in a closed process. However, small- and medium-sized plants use batch processes rather than continuous processes and have adsorption towers installed to prevent air pollution rather than expensive RTOs, despite the fact that they have lower efficiencies. Workers are exposed to these chemicals in the facilities (reactor, mixer). Moreover, with an increase in the use of volatile chemicals, there is an increase in atmospheric emissions. The typical processes taking place during a batch process is studied, using is a paint manufacturing process as an example [13].

In this study, two paint manufacturers were selected. The plant manufactures both resin and paint. Toluene, xylene, and methyl ethyl ketone are all used as solvents, and on average, about 1500 tons per year are used. It is a midsize company with annual sales of 8 billion dollar. The use of solvents in the paint manufacturing process is similar to any paint company, and trade secrets are largely divided into the additives they add. Generally, the paints manufactured is mixed with solvents such as toluene, xylene, methyl ethyl ketone, ethyl acetate, and pigments or resins in a container equipped with a high-speed agitator. The solvents and additives are mixed and boiled in a reactor at a high temperature to produce a resin. This resin is sold by itself or it is processed by secondary processing to produce paint. The solvent may be used to produce a lacquer or a sealant. Depending on the type of paint produced, it is manufactured separately for automobile, ship, building and general industry.

**Figure 1:**
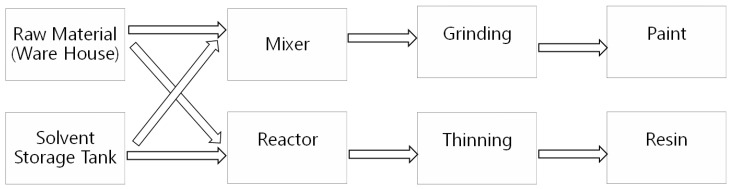
Paint manufacturing process flow chart.

There are two routes in the process flow, either through a reactor or through a high-speed mixer. The step-by-step procedure of the processes are briefly shown in Figure 1.

To estimate the TRI emissions, the process should be classified, and the chemicals released from the process must be estimated. For example, when transporting chemicals from a toluene storage tank to a reactor, the estimates obtained vary, depending on whether it is transported directly to the piping or portable tank. Although it is efficient to transport via pipelines in small-scale production processes, it is reasonable to use them as transport tanks according to the mixing ratio in a paint factory that manufactures various kinds of paint [14].

**Figure 2:**
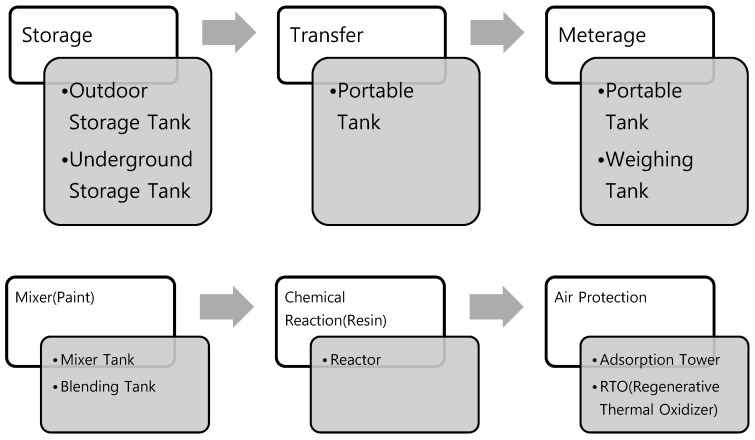
Detailed classification of processes for estimating Toxic Release Inventory emissions.

Processes for calculating TRI emissions from paint plant are classified as follows (Figure 2).

### 2.3. TRI Emission Method

There are four major methods for estimating volatile chemicals in TRI emissions, namely the Emission Factor Method, the Material Balance Method, the Source Testing Method, and the Emission Model Method. Estimating TRI emissions requires conservative results. This is especially true because they are exposed to the community, open to the public, and related to their health. TRI emissions are difficult to estimate accurately. There are many methodologies, but nothing can be said to be the most accurate and realistic. The material balance method is intuitive and simple, but it does not take into account the high volatiles emitted during the process, compared only before and after. Although the source testing method is a direct measurement, it does not record 24 h, so there is a high risk of being overstated or underestimated to estimate annual emissions by instant measurement. The emission factor method does not take into account the volatile characteristics of the chemical itself in detail and has the disadvantage of simply applying the plant operating time and the concentration of the substance. Due to the limitation of modeling. The emission model method may produce different results. However, it is necessary to present the most conservative method compared to each of the four methods and develop a policy to reduce emissions based on the results. This helps to protect the health of local residents and contribute to the protection of the environment. There is another method which involves the replacement of exhaust gas, such as CO or SOx. However, the most popular methods remain the aforementioned four methods. In the guidelines for estimating TRI emissions from foreign countries, including Korea, the entire plant can be analyzed in one way or by using a mixture of methods. According to the 2007 survey, the most common method for estimating Korea’s TRI emissions is the material balance method, as shown in Table 3 [15]. These methods are applied differently for each process, but they are not regulated by the current law. For example, in a point source pollution source, source testing method is more appropriate than material balance method or emission factor method. In this case, the reliability of the results obtained for the estimation of TRI emission is reasonable.

#### 2.3.1. Source Testing Method

This method is used to estimate the amount of TRI emissions based on direct measurement of the actual discharge (flow rate, concentration). If the flow or concentration is not constant and the change is large, the total annual amount of TRI emissions should be estimated by measuring the emission amount every month. This method is effective in estimating the amount of chemical substances emitted from point sources, such as chimneys and wastewater treatment plants [16,17].

**Equation** **1.** *Source testing method calculation formula.*

Source Testing Method Total Emission

$$\begin{array}{c} \mathrm{Emissions}=\mathrm{Discharge}\;\mathrm{average}\;\mathrm{flow}\;\mathrm{rate}\times \mathrm{Average}\;\mathrm{concentration}\\ \times \mathrm{Annual}\;\mathrm{working}\;\mathrm{hours} \end{array}$$

Temperature correction formula

$$\begin{array}{l} \mathrm{Standard}\;\mathrm{Temperature}\;\mathrm{Emissions}\\ \;\;\;\;\;\;\;\;\;=\;\mathrm{Measured}\;\mathrm{Temperature}\;\mathrm{Emission}\times \frac{20\;{}^{\circ}C+273\;{}^{\circ}C}{\mathrm{Measured}\;\mathrm{temperature}\;{}^{\circ}C+273\;{}^{\circ}C} \end{array}$$

Volume Unit $\left(\mathrm{mL}/m^{3}\right)$ Conversion Formula

$$\begin{array}{c} \mathrm{Gas}\;\mathrm{Emission}=\mathrm{Gas}\;\mathrm{Concentration}\;\left(\mathrm{ppmv}\right)\times \;\mathrm{Standard}\;\mathrm{Gas}\;\mathrm{Emission}\;\left(m^{3}/\mathrm{hour}\right)\\ \times \frac{273\times M}{\left(S+273\right)\times 22.4\times 10^{6}}\times \mathrm{Annual}\;\mathrm{working}\;\mathrm{hours} \end{array}$$

M: Molecular Weight, S: Standard temperature.

The measuring instrument used was Minirae 3000 by RAE Systems, a VOC measuring instrument, and IQ-610Xtra, manufactured by wolf, a certification of the Ministry of Environment, Korea.

The instrument for measuring the concentration of toluene, xylene and methyl ethyl ketone is a direct reading instrument. At the moment of measurement, the concentration is indicated on the instrument itself. In this study, the average of the results obtained by measuring end caps, flanges, and valves of pipes was calculated. TRI was calculated by converting this into an annual leak conversion amount (kg). Measurements were taken three times a week during the actual process.

Air emission facilities such as chimneys were asked to self-measurement company once a month to collect and average the annual data of analyzing and measuring the toxic substances emitted.

#### 2.3.2. Material Balance Method

This method estimates the amount of TRI emissions by using the law of conservation of mass. This method is ideal in the case of chemicals with unified mass of unit and cannot be used when the calculated emission amount falls within the error range of the measuring instrument, such as flow meter. Moreover, Enterprise Resource Planning (ERP) records such as inventory list, entry/exit ledger, and sales records were used in this method. It is efficient to estimate total plant emissions. For flare stacks, use the emission factor method whenever possible [16,17].

**Equation** **2.** *Material balance method calculation formula.*

Material Balance Method Total Emission

$$\begin{array}{ll} \mathrm{Emissions} & =\left(\mathrm{Amount}\;\mathrm{introduced}+\mathrm{Amount}\;\mathrm{produced}\;\mathrm{by}\;\mathrm{the}\;\mathrm{reaction}\right)\\ & -\left(\mathrm{Amount}\;\mathrm{leaked}+\mathrm{Amount}\;\mathrm{consumed}\;\mathrm{in}\;\mathrm{the}\;\mathrm{reaction}\right) \end{array}$$

Emissions from each VOC containing material

$$E_{material}=V\times \left(1-\frac{R}{100}\right)\times \left(1-\left[\frac{K}{100}\times \frac{J}{100}\right]\right)$$

$$V=U\times \left(\frac{W}{100}\right)\mathrm{or}\;G\times C$$

*E_material_* = Emissions of VOC Material, *U* = Material usage (kg), *W* = VOC Content (%), *R* = VOC Retained on Substrate (%), *K* = Control Efficiency (%), *J* = Capture Efficiency (%), *V* = VOC Content G = Material Usage (L). *C* = VOC Content (kg/L).

#### 2.3.3. Emission Factor Method

It is a method for estimating the emission of similar sources using the statistically calculated average emission value by directly measuring the emissions according to the state of the substance in various processes and devices. It is efficient in estimating the emissions from piping systems (valves, pumps, compressors, flanges, open lines, sampling points, etc.), which are pollution sources. The average emission factor, emission standard emission factor, emission factor by concentration, and emission factor of industry is different from each other. The emission factors for the oil refining industry and general industry are provided in Table 4. These emission factors vary, depending on whether it is fluid, gas, or liquid. Moreover, in the case of refining industry, the liquid is classified into light oil (specific gravity < 34) and heavy oil (specific gravity ≥ 34). In case of general chemicals, it is classified as light oil if the volatility is higher than kerosene (vapor pressure is more than 5 mmHg at 20 °C) [16,17].

**Equation** **3.** *Emission factor method calculation formula.*

Emission Factor Method Total Emission

$$\begin{array}{ll} \mathrm{Emissions} & =\mathrm{Composition}\;\mathrm{ratio}\;\mathrm{of}\;\mathrm{chemicals}\left(\%\right)\times \mathrm{Number}\;\mathrm{of}\;\mathrm{sources}\\ & \times \mathrm{Emission}\;\mathrm{factor}\;\left(\frac{\mathrm{kg}}{\mathrm{Ton}}\right)\times \mathrm{Annual}\;\mathrm{production}\left(\mathrm{Ton}\right))÷100 \end{array}$$

#### 2.3.4. Emission Model Method

This method uses physicochemical properties of the chemicals (vapor pressure, solubility, diffusion coefficient, etc.) and ideal gas state equations. In addition, it helps estimate TRI emissions by using performance indicators, such as process design data (temperature, pressure, facility size, flow rate, reaction time, residence time, etc.), removal rate, efficiency, and production rate of production process or pollution control facility. It is usually calculated using a modeling program. Korea distributes programs, such as TRIWIN [18]. US EPA distributes programs, such as TANKS and WATER9 free of charge to calculate emissions through the modeling of various facilities [19]. When a chemical substance is injected into a tank or a moving container, it is assumed that the substance evaporates from the container and is discharged into the atmosphere. The formula is as follows [16,17].

**Equation** **4.** *Calculation formula of Emission model method for container injection.*

Emission Model Method Total Emission

(1) Emissions=(a×M×V×P×N)∕(760×R×T)

a = Coefficient by injection method (Table 5); V = Injection capacity (m^3^/times); P = vapor pressure (mmHg); M = (g/mol); N=Annual injection (times/yr); R = 0.082 atm · l/(K·mol); T = Absolute temperature (Operating temperature °C + 273).

### 2.4. Selection of TRI Emission Estimation Method by Process

The paint manufacturing process is divided into six processes: storage process, transfer process, metering process, mixing process, reaction process, and air pollution prevention process. Two methods were selected that could be applied to each of the six process to compare the results with each other. The paint manufacturing plant to be studied was selected by two paint companies that manufactured both resin and paint. There are several methods that can be applied to each process. For example, in the storage process, TRI can be estimated by selecting either the emission model method or the source testing method. The method that can be selected according to the process is shown in Table 6.

### 2.5. Theories and Factors of TRI Emission Estimation by Process

#### 2.5.1. Storage Process, Metering Process, Mixing Process

Storage, metering, and mixing processes are all performed at an ambient temperature and atmospheric pressure, except in the case of fixed tanks. These three processes are estimated by two methods, that is emission model method and source testing method. The storage tanks for toluene, xylene, and methyl ethyl ketone storage processes are all outdoor storage tanks, with a volume of 25 m^3^. The volume of the small portable tank used in the weighing process is 1 m^3^, and the average volume of the mixer used in the mixing process is 2.5 m^3^. This basic information was necessary for calculations (Table 7 and Table 8). The source testing method was selected to calculate the annual emission by converting the measured concentrations of toluene, xylene and methyl ethyl ketone into VOCs in the tank during storage and mixing processes [20].

#### 2.5.2. Transfer Process

The transfer process is based on the piping from the storage tanks to the portable tanks. In the paint manufacture process, portable tanks are used for the batch processes, which are placed in the resin manufacturing building as well as the paint manufacturing building, and the solvent in the storage tank is taken into the portable tank as the piping. In this process, emission factor method and source testing method were compared. Emission factor was calculated based on all the emission factors in Table 4, except for the petroleum refining industry; moreover, the VOCs of the pipe flange, valve, and pump were measured. The plant operation time was assumed to be 300 days a year, 8 h a day.

#### 2.5.3. Reaction Process

In the reaction process, resin and other additives are added and reacted at high temperature (120–150 °C) in a reactor. Resins are used as additives in the paint mixing process or sold as individual products. The average volume of the reactor is 5 m^3^ and the reaction process takes about 40 min on average. Applicable methods include material balance method and source testing method. The source testing method is based on the monthly measurement of the inlet concentration of the adsorption tower, which is the air discharge facility, with respect to concentration (kg/m^3^) and the flow rate (m^3^/day) of the generated substances. The material balance method calculates the difference between the concentration of the input raw material and the concentration of output product, assuming that all the substances have been released.

#### 2.5.4. Air Pollution Prevention Process

The air pollution prevention process involves the collection of reacted (solvents) and discharged (VOCs) from each facility, its transportation from the adsorption tower to activated carbon chamber, and its discharge into the atmosphere. Estimation of TRI emissions includes emission factor method and source testing method. Source testing method is carried out once a month or half-yearly by the plant, according to the Air Quality Conservation Act. The annual emission is calculated based on the measured data. The emission factor method collects the chemical substances generated during the processes, such as reaction and mixing in the local exhaust system (with an efficiency of ~80%, although it may differ depending on the facility). It is a method of estimating that the remaining amount except for the final adsorption rate (the efficiency of the equipment is different, but the adsorption tower efficiency is 80% on average) in the adsorption tower is discharged to the atmosphere.

## 3. Results

### 3.1. TRI Estimation Result by Process

The emission model method for each process was modeled by calculating the average value of two factories. In the source testing method, there was a marginal difference between the two factories, but it was not significant enough; moreover, the average of the two plants was converted to annual TRI emissions. The results of each process are shown in Table 9. In the material balance method, as all the solvents reacted with the resin in the resin manufacturing process, the value obtained during the reaction process is zero. In addition, the emission factor method of the air pollution prevention facility is calculated assuming that the TRI emissions generated by the emission model method of each process are transferred to the adsorption tower. The collection efficiency of process was 80% at metering and mixing, and the reaction process. Storage process’s collection efficiency is 100%. The final collection efficiency of the air pollution prevention facility, which is a point pollution source, was 80%. This can be increased up to 99% with the use of RTO and flare stacks. In pipeline transportation, it is not collected in an air pollution prevention facility but discharged as a non-point pollution source.

### 3.2. Application Method by Process

Based on the results of the study, the application method for each process and substance is presented. The purpose of this study is to evaluate the applicability of the TRI emission estimation method. The emission model method gave better results than source testing method. Although the source testing method can be accurate, it does not measure and monitor continuously. The material balance method was found to be the most used method, unless there was a reaction with the raw materials, which exhausted it and created a blind spot, leading to the emission amount being calculated as zero.

In the case of non-volatile solid materials, material balance method was ideal. However, since chemical substances, such as toluene, xylene, and methyl ethyl ketone volatilize at high temperatures during the reaction process, it becomes redundant. The results of all method may be exaggerated and underestimated. However, it is desirable to develop a conservative outcome method, disclose it to the public, and establish a long-term emission reduction plan [21].

Based on the results of this study, the application methods of each process in the paint manufacturing process are shown in Table 10.

## 4. Discussion

In this study, VOCs, such as toluene, xylene, and methyl ethyl ketone were divided into six processes (storage, transfer, metering, mixing, reaction, and air pollution prevention) and their amounts were estimated by various method. It is difficult to accurately estimate the amount of chemical emissions from the plant to air, water and soil. In particular, emissions to the surrounding air are more difficult to estimate. However, many countries around the world are trying to investigate the type and amount of chemical emissions from plants to formulate emission reduction plans and industrial regulatory measures.

There are a variety of methods for estimating TRI emissions, with changes in each method depending on the physicochemical properties of the chemicals.

In the case of emission model method, the vapor pressure in the factor to be calculated is very high, resulting in high emissions of chemicals with high vapor pressure. Conversely, most heavy metals are not emitted. However, in many countries, heavy metals are emitted from plants in the form of dust, especially in metal manufacturing and mining industries. These processes can be problematic when using the emission model method.

Also, when using the emission factor method in piping, the physicochemical properties of the chemical are not considered, but the emission factor and the operation time are calculated by the number of the flanges and valves. Among the physicochemical properties, heavy oil, light oil, and gaseous substances are distinguished, but even among the light oils, there are many chemical substances with large differences in vapor pressure. Moreover, methyl ethyl ketone has a relatively higher vapor pressure than xylene and toluene. In fact, methyl ethyl ketone is the most abundant chemical found in the source testing method of the transfer process. However, when calculating using the emission factor method, piping lines having the same valve as the tanks will have the same value regardless of the physicochemical properties of the chemical [22].

Furthermore, the material balance method can easily estimate the TRI emission amount, as it assumes that the difference between the chemicals from the plant and the chemicals from the product is emitted during the process. Many plants estimate emissions by the material balance method and submit them to the Ministry of Environment in Korea [15]. In the case of the reaction process in the paint manufacturing plant, the result obtained is zero. This is because the total amount of the solvent is converted to resin; thus, no solvent is present in the product. Source testing method is the most realistic approach. Some advanced plants have systems that measure 24 h discharge concentrations using the Leak Detection and Repair (LDAR) system [23]. Other plants have a system that monitors the control room by attaching a measuring device to each section. This is because in the case of non-point sources, such as pipes, it is difficult to predict which section will leak [24]. However, not all factories can use these methods, especially since measurements in pipelines are a one-time measure there is no guarantee whether the concentration has been low throughout the year, or low only on the particular day of measurement due to no leakage. Source testing method is valid for air pollution prevention facilities because periodic pollutants, such as adsorption towers and scrubber RTO are measured periodically (1 week, 1 month, half-yearly) and the final emission concentration is controlled [25].

In addition, physicochemical properties of the chemicals used in the measurement of TRI emissions should be considered. Methyl ethyl ketone has a vapor pressure that is 3 to 10 times greater than that of toluene and xylene (Table 7). In emission model method, the actual vapor pressure has a large effect on the model, resulting in TRI emissions that show methyl ethyl ketone having a twice as large value as compared to the other materials. Even though the actual value (source testing method) of methyl ethyl ketone is large when measured directly, the difference is small compared to the emission model method.

The emission factor method estimates TRI emissions based on the results emission model method. Therefore, if the differences in emission model method are large, an exaggerated value can be found in the emission factor method. On the other hand, the results of the actual adsorption tower measurement showed that toluene and xylene had a larger source testing method than the emission model method.

This is likely to affect the management and influence of plant air pollution prevention facilities. Moreover, the cycle of replacing the activated carbon installed on the adsorption tower differs from place to place. If it is time to replace but has not been replaced, its efficiency may be lower than 80% (emission factor method), which may actually discharge a large amount of chemicals [26].

The emission factor method of the transfer process assumes a high volatility if the vapor pressure is 5 mmHg or more at 20 °C [27]. It is also necessary to select the reference vapor pressure in the air pollution prevention process.

Thus, it is necessary to select a combination of processes of each plant and the characteristics of the chemicals to be handled. Each plant needs to review the appropriateness of the method currently underway. At present, the feasibility assessment of the TRI emission method has not been done in Korea. This study is expected to contribute to the selection of the emission method of paint manufacturing plants and to the accuracy and conservative results of TRI emissions. In the short term, efforts should be made by both national and industrial sectors to choose people’s right to know and policies to prevent environmental pollution and, in the long term, to reduce emissions for the betterment of public health.

## 5. Conclusions

This study focused on the estimation of chemical TRI emissions in plants and the global feasibility of the method. OECD member countries have an obligation to investigate the amount of chemicals released and to disclose them to the public [28]. Moreover, it necessary to establish regional reduction plan for areas with large emissions of toxic chemicals and to notify the residents about their risks [29].

Among the various chemicals, volatile chemicals have been studied mainly in paint factories, which are relatively vulnerable compared to other industries. As a result, various methods for estimating the amount of TRI emissions have been developed for each chemical by process. The material balance method underestimates values in the reaction process, and the emission factor method exaggerates results in the transfer process. Moreover, source testing method has a limit of one-time measurement (Table 9).

Nonetheless, the risks need to be approached conservatively. Paint manufacturing plants should choose a method that produces more TRI emissions among the different methods. As a result, it is appropriate to evaluate the storage process, weighing process, and mixing process by an emission model method. Source testing method risks underestimating annual emissions. The process of transferring to piping should be evaluated by the emission factor method. Source testing method in pipes can result in much underestimated results, especially because it is difficult to pinpoint leaks. At the plant, care must be taken to ensure that there are no leaks at the joints, such as the flange or valves of the pipe. The reaction process and the air pollution prevention process should, if possible, estimate the emissions based on directly measured results (source testing method). The material balance method in the reaction process has the advantage of being simple to use. However, emissions of toxic chemical emitted in the course of the reaction are not considered. Since only the results before and after the reaction are compared, there is a problem that does not take into account the amount of toxic chemicals emitted by the volatiles in the reactor reacting at high temperature. However, even in the case of air pollution prevention process, it is appropriate to calculate the emission factor method for methyl ethyl ketone. Thus, it is necessary to estimate the method in with respect to the physicochemical properties of the chemical as well as the process.

Through this study, we can estimate the TRI emission method of paint manufacturing process. However, it is also necessary to continuously evaluate the feasibility of the method with respect to chemical substances and processes used in other industries. This study is limited in that it focuses on volatile chemicals among various toxic chemicals. In addition, among other various manufacturing plants, the progress of the paint manufacturing plant is limited. Subsequent studies may proceed with processes that deal with potentially accidental and dangerous alkalis and acids among the chemicals used in industry such as hydrochloric acid and nitric acid.

## Figures and Tables

**Table 1:** Ratio of chemical emissions country-wise in 2009.

Rank	The United States	Japan	Australia	Korea
1	Hydrochloric acid (23.4%)	Toluene (42.2%)	Ammonia water (22.1%)	Xylene (28.1%)
2	Sulfuric acid (6.4%)	Xylene (16.7%)	Hydrochloric acid (12.4%)	Toluene (15.6%)
3	Methanol (6.3%)	Methyl ethyl ketone (8.7%)	Methanol (8.7%)	Ethyl acetate (9.5%)
4	Toluene (1.8%)	Dichloromethane (3.1%)	Toluene (6.9%)	Methyl ethyl ketone (7.2%)
5	Xylene (1.6%)	Ethyl benzene (2.9%)	Xylene (5.5%)	Dichloromethane (5.5%)

% is the ratio of the total chemical substances emitted. Table 1. From. Ahn sun chan; hong seok il. Comprehensive Assessment of Chemical TRI Emissions System and Development of Future Development Plan. 1st ed; Ministry of environment: Seoul, Republic of Korea, 2009; pp. 22–57 [10].

**Table 2:** Ratio of amount chemical substances released route by country in 2007.

Rank	The United States	Japan	Australia	Korea
1	Air (61.6%)	Air (95.1%)	Air (98.1%)	Air (99.6%)
2	Water (10.9%)	Water (3.9%)	Water (1.7%)	Water (0.03%)
3	Soil (27.5%)	Soil (1.0%)	Soil (0.11%)	Soil (0.01%)

Table 2. From Park hyun soo. Prepare a plan to improve the chemical TRI emission survey system. 1st ed; Ministry of environment: Seoul, Republic of Korea, 2007; pp. 6–45 [12].

**Table 3:** Ratio of amount chemical substances released route by Korea in 2007.

Method	Ratio (%)
Emission Model Method	10
Emission Factor Method	4
Material Balance Method	47
Source Testing Method	8
Mixture of methods	31
Sum	100

Table 3. From Gong sung yong; Lee sang mok. Environmental Forum: Achievements and Challenges of Chemical TRI Emissions Inspection System. Environmental Forum 2010, vol 164, pp. 1–8 [15].

**Table 4:** Emission factor for industry.

Source	State	Emission Factor
		(kg/h/source)
Valve	Gas	0.00597
	Light oil	0.00403
	Heavy oil	0.00023
Pump	Light oil	0.0199
	Heavy oil	0.00862
Compressor	Gas/steam	0.228
Safety valve	Gas/steam	0.104
Connector (flange, manhole)	All	0.00183
Open lines	All	0.0017
Sampling points	All	0.0150

Table 4. From National Institute of Chemical Safety. Guidelines for the investigation of chemical TRI emissions; Ministry of environment; Daejeon, Republic of Korea, 2019; pp. 13–41 [17].

**Table 5:** Coefficient for injection conditions.

Injection conditions	Coefficient (a)
Empty tank, infusion under the face	0.5
Empty tank, spraying on top of liquid	1.45
Normal state, infusion under the face	0.6
Normal state, spraying on top of liquid	1.45
Normal state, infusion under the face with pressure control	1.0
Normal state, spraying on top of liquid with pressure control	1.0

**Table 6:** Selection of TRI emission estimation method for each process.

Storage Process	Transfer Process	Metering Process	Mixing Process	Reaction Process	Air Pollution Prevention Process
Emission Model Method	Emission Factor Method	Emission Model Method	Emission Model Method	Material Balance Method	Emission Factor Method
Source Testing Method	Source Testing Method	Source Testing Method	Source Testing Method	Source Testing Method	Source Testing Method

**Table 7:** Physicochemical characteristics of chemicals.

	Toluene	Xylene	Methyl Ethyl Ketone
CAS No (Chemical abstracts service registered number)	108-883	1330-20-7	78-93-3
Molecular weight	92.14	106.16	72.11
Density (kg/m^3^)	805	867	864
Vapor pressure (mmHg at 25 °C)	28.4	6.65	90.6

**Table 8:** Basic specifications and operating conditions of tank.

	Storage Tank	Weighing Tank	Mixing Tank
Volume of tank m^3^	25	1	2.5
Diameter, Height mm	(D ^1^: 3100, H ^2^: 4500)	(D ^1^: 500, H ^2^: 575)	(D ^1^: 1000, H ^2^: 1200)
Tank color coefficient	1, 2 ^3^	1, 2 ^3^	1, 2 ^3^
Number of injections per year	50	1250	1250
Absolute temperature in tank (K)	298	298	298
Coefficient according to injection condition	Empty tank, infusion under face	Empty tank, spraying on top of liquid	Normal state, infusion under the face
	0.5	1.45	0.6

^1^ Diameter, ^2^ height, ^3^ in the case of silver 1.2; white is 1.0; pale green 1.36; Others 1.44.

**Table 9:** Annual TRI emission results by process (kg/year).

TRI Emission (kg/year)	Toluene	Xylene	Methyl Ethyl Ketone
Storage process Emission Model Method	210.7	62.1	503.0
Storage process Source Testing Method	106.3	12.25	83.2
Metering process Emission Model Method	101.6	27.5	214.8
Metering process Source Testing Method	54.3	6.3	42.5
Mixing process Emission Model Method	118.3	53.9	627.3
Mixing process Source Testing Method	27.1	31.3	212.5
Transfer process Emission Factor method	571.3	559.2	535.1
Transfer process Source Testing Method	93.8	81.4	123.7
Reaction process Material Balance Method	0	0	0
Reaction process Source Testing Method	488.4	382.2	562.7
Air pollution prevention process Emission Factor method	77.3	25.4	235.3
Air pollution prevention process Source Testing Method	86.8	67.9	100.1

**Table 10:** Method for estimate TRI emissions by process.

	Storage Process	Transfer Process	Metering Process	Mixing Process	Reaction Process	Air Pollution Prevention Process
Toluene	A	C	A	A	D	D
Xylene	A	C	A	A	D	D
Methyl ethyl ketone	A	C	A	A	D	C

A: Emission model method, B: Material balance method, C: Emission factor method, D: Source testing method. There are four method to estimate TRI, but Material balance method was not applied in any process.
